# Comparison of Continuous Glucose Monitoring between Dexcom G4 Platinum and HD-XG Systems in Nonhuman Primates (*Macaca Fascicularis*)

**DOI:** 10.1038/s41598-017-09806-w

**Published:** 2017-08-29

**Authors:** Bingdi Wang, Wei Qiao, Weiwei Ye, Xiaoli Wang, Yongqiang Liu, Yixin (Jim) Wang, Yong-Fu Xiao

**Affiliations:** Crown Bioscience, Inc., Taicang, Jiangsu Province China

## Abstract

Timely knowing glucose level helps diabetic patients to manage the disease, including decisions about food, physical activity and medication. This study compared two continuous glucose monitoring systems in conscious and moving-free nonhuman primates (NHPs, *Macaca fascicularis*). Each normoglycemic or diabetic monkey was implanted with one Dexcom G4 Platinum subcutaneously or one HD-XG glucose sensor arterially for glucose monitoring. The glucose levels measured by both telemetry devices significantly correlated with the glucometer readings. The data of oral glucose tolerance test (oGTT) showed that the glucose levels measured by either Dexcom G4 Platinum or HD-XG transmitter were very similar to glucometer readings. However, compared to HD-XG transmitter or glucometer, Dexcom G4 Platinum detected a decreased glucose peak of ivGTT with approximately 10 min delay due to interstitial glucose far behind blood glucose change. Our data showed the advantages of the telemetry systems are: (1) consecutive data collection (day and night); (2) no bleeding; (3) no anesthesia (moving freely); (4) recording natural response without physical restriction and stress; (5) less labor intensity during ivGTT and other tests; (6) quick outcomes without lab tests. This article summarized and compared the differences of the general characteristics of two continuous glucose monitoring systems in diabetic research.

## Introduction

Dysfunctional carbohydrate metabolism without treatment eventually leads to diabetes which significantly impacts on the quality of patient life. Potential new therapies and technologies may help to improve the quality of life beyond current standard of care and perhaps even to cure the disease in future^1, 2^. Various animal models have been used in research for understanding the disease and discovering new novel therapies^3–6^. Nonhuman primates (NHPs) can naturally develop type II diabetes mellitus (T2DM) in a way similar to the onset and progression of T2DM in humans, which makes them an excellent model for diabetes and obesity research^7–12^.

Glucose monitoring helps diabetic patients manage the disease to avoid its associated complications^13, 14^. Pricking a fingertip with an automatic lancing device to obtain a blood sample and to measure the blood sample’s glucose level by glucometer is the most common way to check glucose level^15^. Dexcom G4® PLATINUM (Dexcom, Inc., San Diego, CA, USA) was approved by the U.S. Food and Drug Administration (FDA) for continuous glucose monitoring (CGM) in diabetic patients. The transmitter securely sends out vital glucose information for real-time diabetes management without pricking finger, which is making diabetes management more convenient and flexible than ever before^13^.

Another telemetry system, HD-XG implantable glucose device (Data Sciences International, Saint Paul, MN, USA), provides continuous measurement of blood glucose, temperature and locomotor activity for up to 2 months in rodents and NHPs^16, 17^. The system has an electrochemical glucose oxidase sensor placed in one main artery for real-time measurements of blood glucose plus temperature and physical activity. Continuous glucose monitoring via implanted telemetry device is very limited in biomedical research, especially in large animals, because blood glucose is often measured by handheld glucometer, clinical chemistry analyzer or Analox analyzer. These conventional methods require sampling blood periodically, which may induce stress and lose blood volume obviously in small animals if bleeding frequently, as well as possibly miss some critical data points during sampling intervals, especially night time. Thus, ‘around the clock’ measurements of blood glucose in unstressed, moving-free animals have unique advantages in research. This study summarized the advantages and some characteristics of using Dexcom G4® Platinum and HD-XG transmitter systems to monitor body glucose continuously in conscious, stress-free cynomolgus monkeys (*Macaca fascicularis*) with or without diabetes.

## Materials and Methods

**Figure 1:**
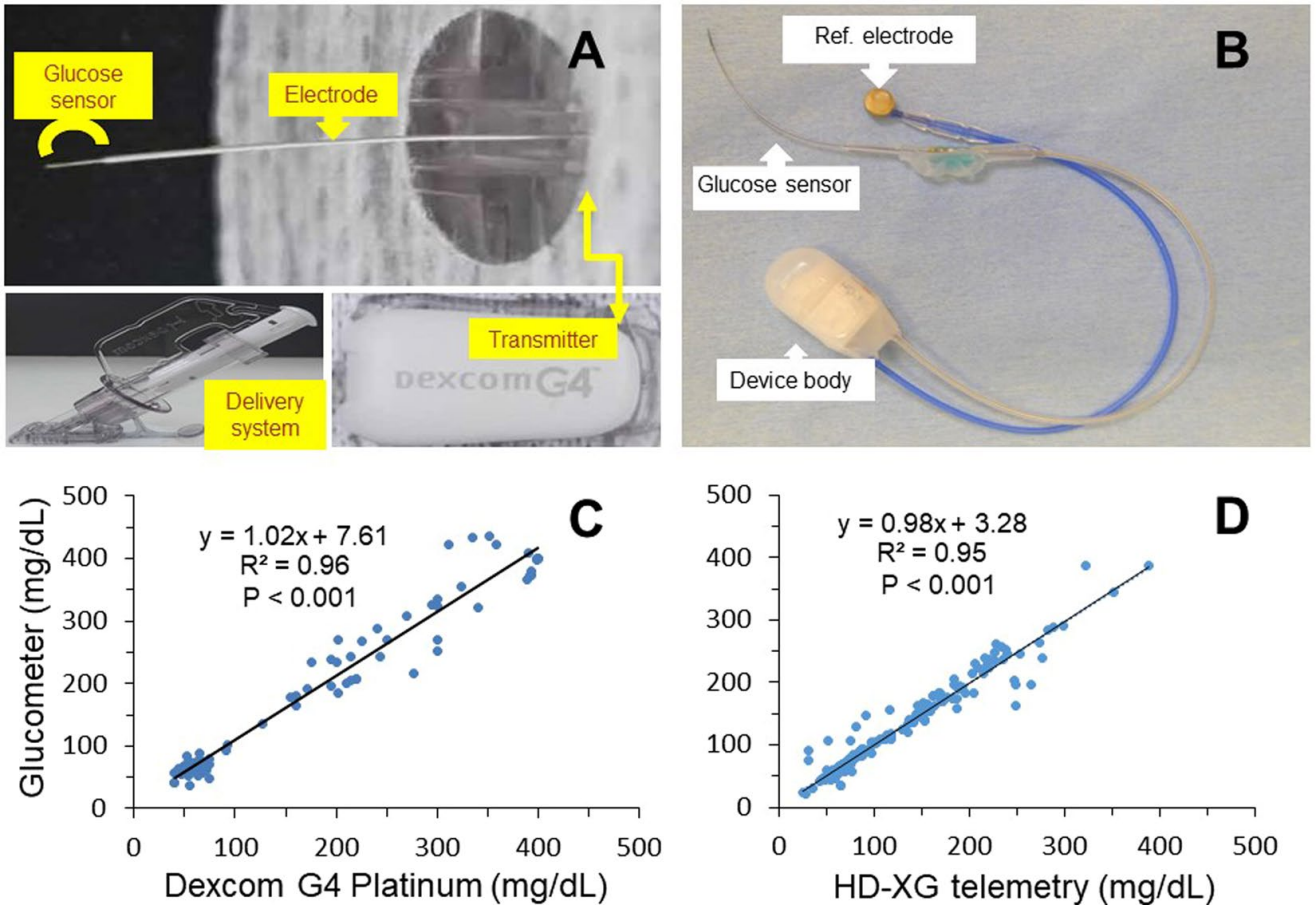
(**A**) FDA-approved Dexcom G4 Platinum sensor for continuous glucose monitoring. The free tip was an 0.12 mm diameter platinum electrode (Glucose sensor) for sensing glucose after subcutaneous implantation and the other end was connected to a transmitter placed on the skin surface. The inserts show a sensor delivery syringe loaded with one glucose sensor ready for subcutaneous implantation and a signal transmitter. (**B**) Implantable HD-XG telemetry device for continuous monitoring of glucose, temperature, and locomotor activity. (**C**) Significant correlation between the glucose levels measured by Dexcom G4 Platinum and glucometer. (**D**) Significant correlation between the blood glucose levels measured by HD-XG telemetry device and glucometer.

The Dexcom G4 Platinum system was obtained from Dexcom, Inc. (San Diego, CA, USA) and included sensor, transmitter and receiver, as well as computer and software for data collection and analysis. The sensor was a small piece of wire (Fig. 1A) that was inserted into the skin via the delivery system for measurement of interstitial fluid glucose level every 5 minutes for up to 7 days. The measured glucose signals were sent through the transmitter to the receiver where they were displayed and recorded for analysis. The HD-XG glucose telemetry system was provided by Data Sciences International (DSI, Saint Paul, MN, USA), including one HD-XG transmitter (Fig. 1B), intermediate transmitter, signal receiver, computer and software for data collection and analysis.

### Animals and animal care

Experiments were performed in normoglycemic and diabetic cynomolgus monkeys of either sex (Table 1). The diabetic monkeys were selected from spontaneously developed diabetic ones who met the diabetes criteria for experimental monkeys and were housed in our animal facility^7^. These monkeys were individually housed and maintained in our animal facility in accordance with the guidelines of the Association for Assessment and Accreditation of Laboratory Animal Care (AAALAC). The room temperature was maintained at ∼21 °C with a 12 hr light/dark cycle with lights off from 7 PM to 7 AM. All the animals were free access ad libitum to water and a complete, nutritionally balanced normal diet (Beijing Keao Xieli Feed Co., LTD, Beijing, China) enriched with seasonal fruit and vegetables. The experimental protocol was approved by the Institutional Animal Care and Use Committee (IACUC) of Crown Bioscience, Inc.

**Table 1:** Characteristics of the cynomolgus monkeys enrolled in the experiment.

Group	N	Age (year)	BW (kg)	Glucose (mg/dL)	Insulin (µIU/mL)	HbA1c (%)
**Dexcom G4**						
Normoglycemia	5	14.2 ± 1.4	6.7 ± 0.2	78 ± 2	21 ± 5	4.6 ± 0.3
Diabetes	5	16.0 ± 1.8	9.0 ± 0.7*	210 ± 17***	36 ± 24	10.3 ± 1.3*
**HD-XG**						
Normoglycemia	3	7.3 ± 1	6.2 ± 0.9	58 ± 3	28 ± 13	4.7 ± 0.4
Diabetes	*a*	17	5.9	120	9.6	4.7
	*b*	16	7.1	114	30.3	5.9

Note: Values are expressed as mean ± SEM, except those in HD-XG diabetes group which shows the single individual data of animal *a* and animal *b*. BW, body weight. *p < 0.05; ***p < 0.001; vs. normoglycemia.

To obtain the parameters showed in Table 1, each overnight-fasted (approximately 16 hrs) monkey was placed in a monkey chair. The body weight was measured and recorded. Blood (2 mL/each) was collected from a cephalic or saphenous vein into two K2-EDTA tubes which were immediately placed on ice. The animal was returned to its cage after blood collection. One sample tube was gently inverted for 10 times and plasma was separated by centrifugation at 3000 rpm for 15 min at 4 °C. The plasma was then stored at −80 °C until analysis. Another blood sample was kept at 4 °C for glycated hemoglobin (Hb1Ac) assay. All the samples were analyzed within 3 to 4 hrs after blood collection. Blood HbA1c (by ion-exchange HPLC), plasma glucose (by Siemens Advia-2400) and insulin (by Siemens Advia Centaur XP) were analyzed at the clinical lab (The First People’s Hospital of Taicang, Taicang, Jiangsu Province, China).

### Implantation of Dexcom G4® PLATINUM

Following the instruction of the Dexcom G4 Platinum Professional System (Dexcom Inc., San Diego, CA, USA), its receiver was fully charged one day before sensor insert and then set the time, date, transmitter ID and glucose alert range. On the day of sensor insertion, each experimental monkey with approximately 16-hr fasting was intramuscularly administered with ketamine at 10 mg/kg (Fujian Gutian Pharmaceutical Co. Ltd., Fujian, China). The skin around the upper back of the animal was shaved and aseptic with betadine and then 70% ethanol. The sensor was inserted into the skin with the injection device and then was connected with a transmitter (Fig. 1A). The transmitter was placed on the local surface of the skin and fixed with bandage. A monkey jacket was then placed on after device fixation.

### Implantation of HD-XG telemetry

An implantable HD-XG telemetry device (DSI, St. Paul, MN, USA) consists of one glucose sensor lead, one reference lead and device body (Fig. 1B) and is packed and sterilized ready for use. The operation room and surgical tools were disinfected one day before surgical operation. On the experimental day for device implantation, each overnight-fasted monkey received ketamine (15 mg/kg, Fujian Gutian Pharmaceutical Co. Ltd., Fujian, China) and 0.01 mg/kg buprenorphine by intramuscular administration with additional ketamine (5 mg/kg) as needed during operation. Body temperature was monitored and maintained at ~37 °C by a thermostatically controlled warm water–circulating pad placed beneath the body. The skin around the intended incision site was shaved and aseptic with betadine starting from the center of the incision region to spiraling outward. Then, the area was swept with 70% ethanol gauze pad. The vital signs, such as heart rate, blood oxygen saturation and respiration rate, were monitored during surgery. A small incision was made in the femoral area and one branch from the femoral artery was carefully dissected. The glucose sensor electrode of the HD-XG device was cannulated into the artery branch until its tip in the main femoral artery and then ligated the artery branch together with the sensor electrode. The reference electrode and device body were fixed subcutaneously in the place near by the femoral artery. The incision was then sutured and covered properly with gauze. The monkey was placed in a monkey jacket and an intermediate transmitter was left in the jacket pocket^17^.

After implantation of either a Dexcom G4 Platinum or a HD-XG device, the monkey was then returned to its housing cage after regaining consciousness. Buprenorphine at 0.01 mg/kg was injected intramuscularly every 6–12 hours after implantation for 2 days and antibiotic amoxicillin at 7 mg/kg was also given intramuscularly if needed. The health of a surgical animal was closely monitored during one-week recovery. Food and water were provided again after the surgical animal was returned back to its housing cage and fully recovered from anesthesia.

### Device calibration

The Dexcom G4 Platinum system detected tissue fluid glucose level via a tiny glucose sensor. A transmitter sent the information about the tissue glucose levels via radio waves from the sensor to a pager-like wireless monitor. The blood glucose levels were checked with the glucometer (One Touch® Ultra®, LifeScan, Inc., Milpitas, CA) via pricking the animal tail at the cage side to obtain blood draping to the glucose test strip and used to program and calibrate the device twice daily. In the meantime, clinical observation of each animal was conducted after insertion of Dexcom G4 Platinum.

To obtain an optimal performance and accuracy of glucose readings, the implanted HD-XG device was calibrated by reference measurements of tail vein blood samples with the Nova StatStrip Glucose Meter (Nova^®^ Biomedical, Waltham, MA, USA) from time to time during the study. Raw telemetry data were recorded in nanoamperes (nA) and calibration reference values were recorded in mg/dL. The calibration algorithm converts the telemetry (nA) data to the values that were equivalent to the appropriate mg/dL results. Multi-point calibrations were needed to establish a linear relationship between the sensor outputs and blood glucose levels at beginning of initial data collection and also at the end of the study. To get multi-point calibrations, blood glucose levels differed by at least 100 mg/dL with intravenous glucose injection to minimize calibration errors due to the inaccuracy of glucose reference. Single-point calibrations with glucometer were also performed at least twice per week to verify baseline glucose value and to prevent non-physiologic changes resulting from sensor drifting due to enzyme instability or fibrin growth on the sensor electrode.

### Data collection

**Figure 2:**
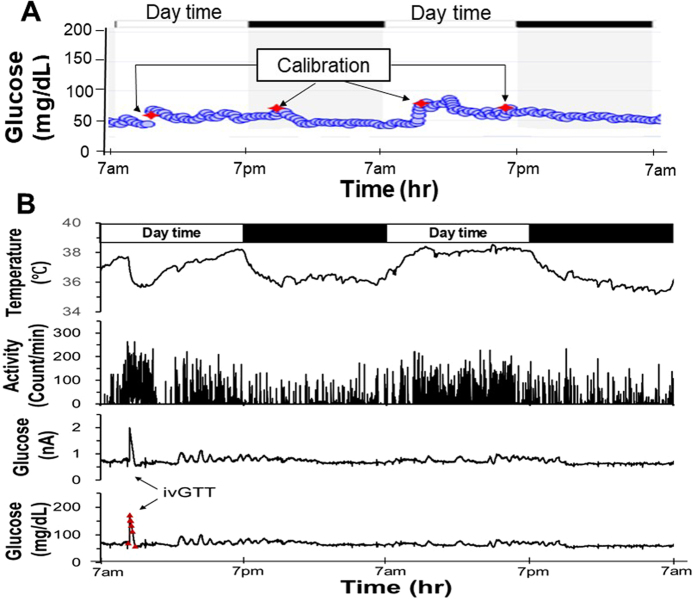
Representative traces collected continuously from one conscious monkey implanted with a Dexcom G4 Platinum and another monkey with a HD-XG device. Panel A The marker on the top showed the day time (open bar) and night time (filled bar). Calibrations (Spots in red) were conducted twice per day during the period of glucose continuous monitoring. More variabilities of glucose levels occurred during the 2^nd^ day resulted from experimental procedures and feeding. Panel B From top to bottom: body temperature (1^st^ trace) with the marker on top to indicate day time (open bar) and night time (filled bar), physical activity (2^nd^ trace), blood glucose electrical signal (3^rd^ trace), and blood glucose level (4^th^ trace) after calculation from the corresponding glucose electrical signal. One ivGTT was conducted in the monkey during the 1^st^ day monitoring (see the arrows).

After calibrations with the blood glucose levels measured by glucometer, interstitial or blood glucose levels were continuously measured by the Dexcom G4 Platinum system (Fig. 2A) or by the implanted HD-XG telemetry system for 24 hrs per day (Fig. 2B). In the meantime, blood glucose levels during oral glucose tolerance test (oGTT) or intravenous glucose tolerance test (ivGTT) were measured by both glucometer and telemetry device. For ivGTT, the animals were overnight-fasted for 16 hrs and anesthetized with an initial dose of ketamine at 15 mg/kg (i.m.) with additional dose during the procedure if needed. A cephalic or saphenous vein was cannulated for glucose infusion. Glucose (50% dextrose, 0.25 g/kg) was intravenously infused during 30 sec and the system was flushed with 5 ml heparinized saline to push the residual glucose into the blood stream. Blood glucose levels were measured by the glucometer via pricking the animal tail at the cage side immediately before and 3, 5, 10, 15, 20, 30 min after glucose infusion and also detected by the implanted HD-XG transmitter or interstitial glucose by a Dexcom G4 device. After ivGTT procedure the animal was returned to its cage under close monitoring until its recovery from anesthesia.

For oGTT, each monkey was overnight fasted for approximately 16 hrs. After being placed in a monkey chair, a nasal/oral gavage tube was placed into the stomach. Glucose 1.75 g/kg in 35% glucose solution was administered via the gavage tube at 5 mL/kg in each conscious animal followed by flashing with 5 mL 0.9% saline to wash the residual glucose solution into the stomach. Blood glucose levels were measured by the glucometer via pricking the animal tail at the cage side immediately before and 15, 30, 60, 90, 120, 180 min after glucose administration and also continuously detected by implanted the HD-XG transmitter or interstitial glucose by the implanted Dexcom G4 device. The animal was placed back to its cage from the monkey chair after completion of oGTT.

In another experiment, to avoid the stress induced by oral gavage, banana-glucose GTT was conducted via naturally feeding in overnight-fasted conscious monkeys. Glucose (1.75 or 0.75 g/kg) was buried in a piece of banana (10 g/kg) to feed the animal for the test. Blood glucose levels were monitored via glucometer immediately before and 15, 30, 60, 120 min after glucose administration and also via Dexcom G4 Platinum (tissue fluid glucose) telemetry continuously for 180 min.

## Results

### Correlation of glucose levels measured by glucometer and telemetry device

To validate the reliability of the telemetry methods, glucose levels were measured at different time points with or without various challenges. During the study, 90 tissue glucose data points were collected by the Dexcom G4 Platinum method and at the same time, 90 blood glucose readings were measured by the glucometer in normoglycemic (n = 5) and diabetic (n = 5) monkeys. The glucose levels measured by the two methods were highly correlated (Fig. 1C, *Y* = 1.02x + 7.61, R^2^ = 0.96, *p* < 0.001), which suggests that the Dexcom G4 Platinum method is reliable for continuous glucose monitoring. In addition, 187 blood glucose parameters were collected by both HD-XG telemetry and glucometer in normoglycemic (n = 3) and diabetic (n = 2) monkeys. The blood glucose levels measured by the telemetry method were highly correlated with the glucometer readings (Fig. 1D, *Y* = 0.98x + 3.28, R^2^ = 0.95, *p* < 0.001), which suggests that the telemetry method with the implantable HD-XG device is also reliable for continuous blood glucose monitoring.

### Glucose measurements by Dexcom G4 Platinum or HD-XG during glucose tolerance test

**Figure 3:**
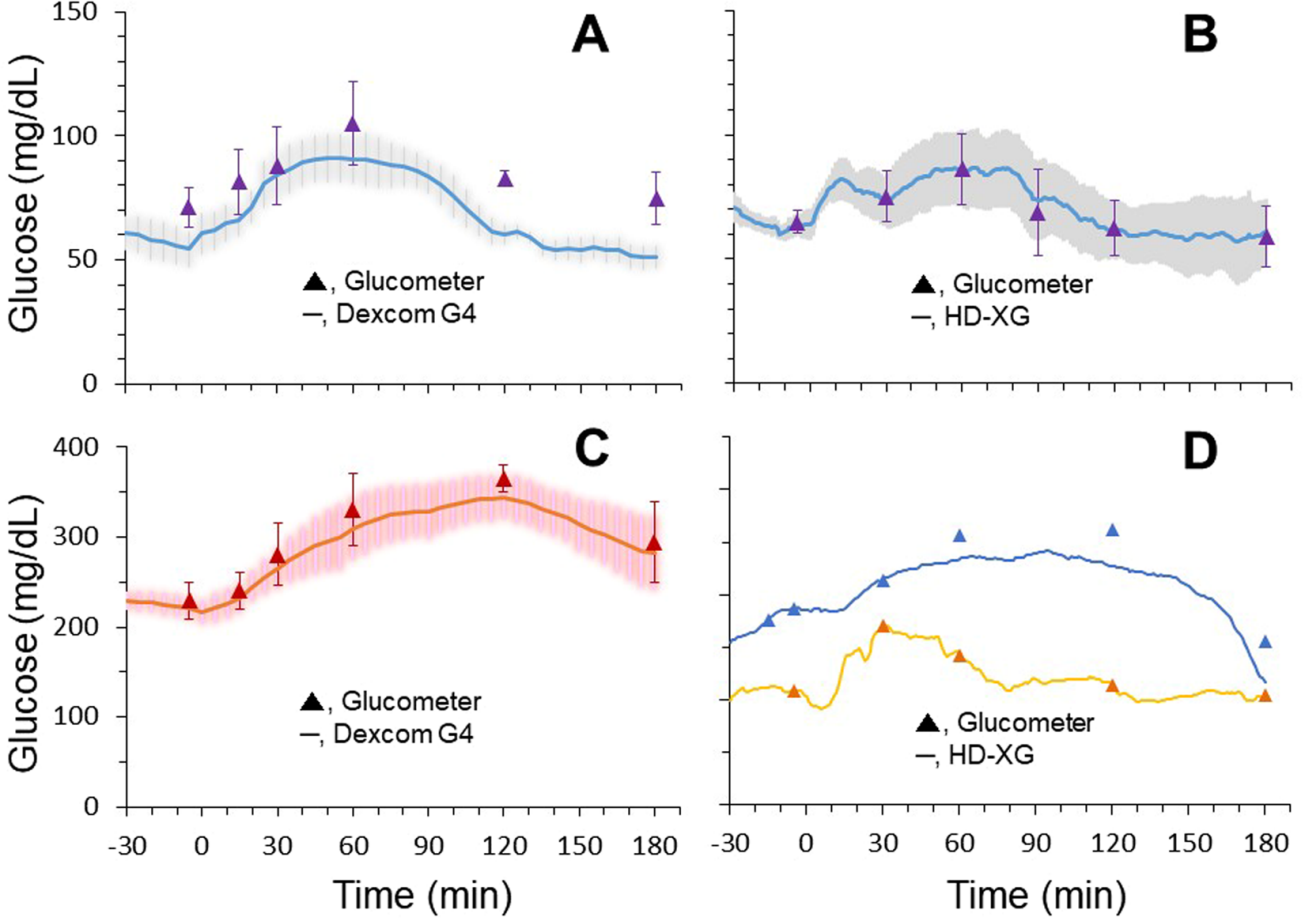
Oral glucose tolerance test (oGTT) was performed in the conscious normoglycemic (Panel A, n = 5 and B, n = 3) and hyperglycemic (Panel C, n = 5 and D, n = 2) monkeys. The glucose levels during glucose gavage oGTT were continuously monitored by the subcutaneously implanted Dexcom G4 platinum devices (tissue glucose, Panel A and C) or by the arterially implanted HD-XG devices (blood glucose, Panels B and D) and also by the glucometer simultaneously (blood glucose, Panel A,B,C and D).

To evaluate the compliance of the Dexcom G4 Platinum and HD-XG methods with the changes of blood glucose, oGTT and ivGTT were performed in the experimental monkeys according to the method reported previously^7, 8, 18–21^. The results obtained from implanted Dexcom G4 Platinum or HD-XG telemetry devices were compared with the readings by the glucometer (StatStrip Xpress meter, Waltham, MA, USA) via the tail prick method. Compared with the glucometer readings, the tissue glucose levels measured by the Dexcom G4 Platinum system were relatively lower during oGTT in both normoglycemic (Fig. 3A, n = 5) and diabetic (Fig. 3C, n = 5) monkeys. In contrast, the blood glucose levels measured by the HD-XG telemetry system were similar to the blood glucose readings tested by the glucometer during oGTT in either normoglycemic (Fig. 3B, n = 3) or diabetic (Fig. 3D, n = 2) monkeys.

**Figure 4:**
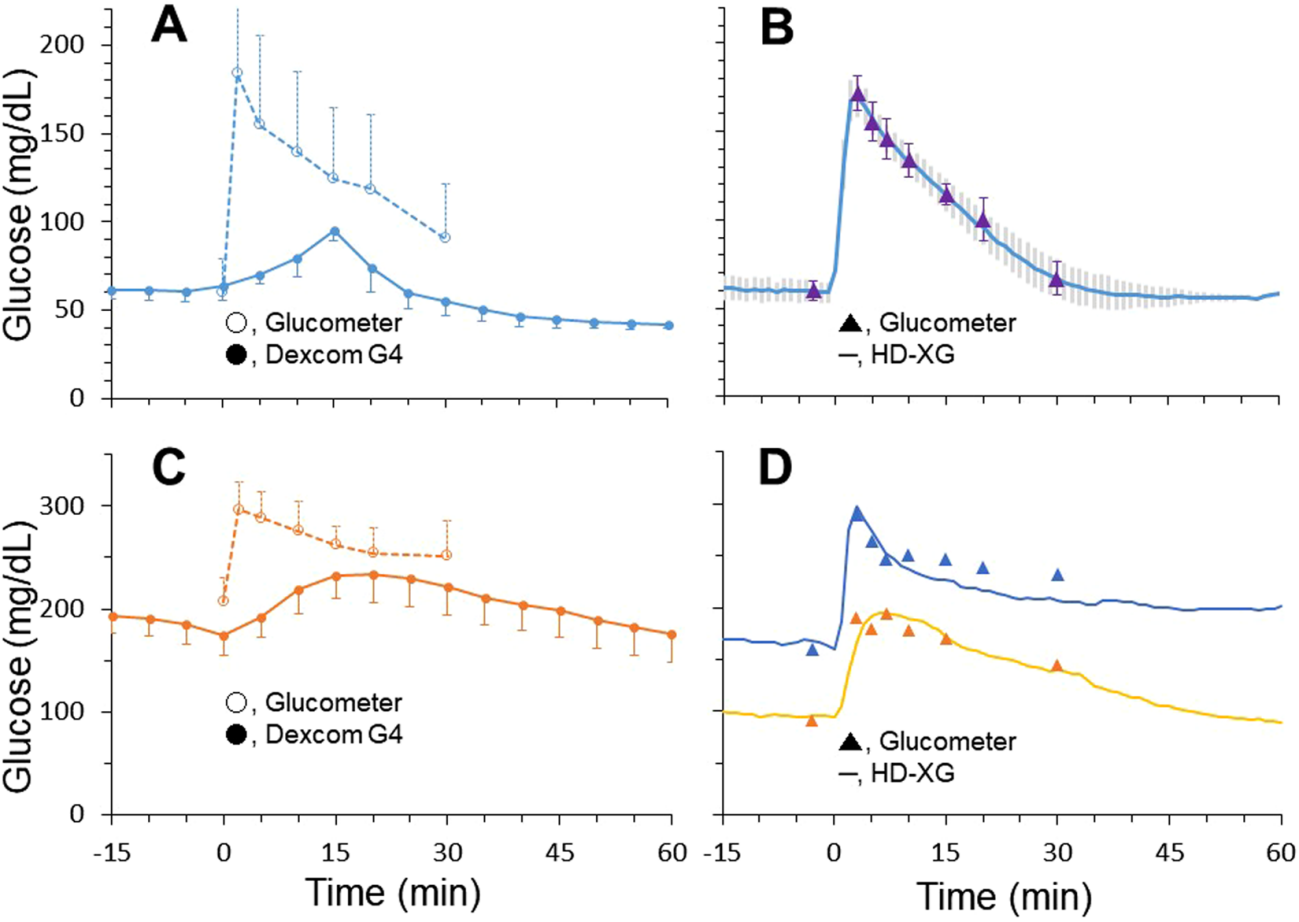
Intravenous glucose tolerance test (ivGTT) was performed in the conscious normoglycemic (Panel A, n = 5 and B, n = 3) and hyperglycemic (Panel C, n = 5 and D, n = 2) monkeys. The glucose levels were continuously monitored by the subcutaneously implanted Dexcom G4 platinum devices (interstatial glucose, Panel A and C) or by the arterially implanted HD-XG devices (blood glucose, Panel B and D) and also by the glucometer simultaneously (blood glucose, Panel A,B,C and D).

In another group of animals, the compliance of glucose measurements by the Dexcom G4 Platinum and HD-XG telemetry methods with glucometer readings were also verified during intravenous glucose tolerance test (ivGTT). After intravenous glucose injection tissue glucose changes were monitored and recorded by the implanted Dexcom G4 Platinum devices (solid circle, •) in normoglycemic (Fig. 4A, n = 5) and diabetic (Fig. 4C, n = 5) for 60 min. At the same time, the blood glucose levels were also tested by the glucometer (StatStrip Xpress meter) via the tail prick method (open circle, ○) immediately before and 3, 5, 10, 15, 20 and 30 min after glucose administration (Fig. 4A and C, n = 5). Compared with the glucometer blood glucose readings, the peak of the tissue glucose measured by the implanted Dexcom G4 Platinum devices was markedly delayed and reduced during ivGTT in both normoglycemic and diabetic monkeys (Fig. 4A,C).

In another group of animals, the blood glucose levels were continuously monitored and recorded by the implanted HD-XG telemetry devices and in the meantime also measured by the glucometer (▲) in normoglycemic (Fig. 4B, n = 3) and diabetic (Fig. 4D, n = 2). The changes of the blood glucose levels measured by the glucometer via the tail prick method immediately before and 3, 5, 7, 10, 15, 20 and 30 min after glucose administration matched well with the results continuously recorded by the implanted HD-XG telemetry devices (Fig. 4B,D). Compared with the glucometer method, the HD-XG device method had no delay and reduction of the peak glucose values during ivGTT in both normoglycemic and diabetic animals.

### Blood glucose response to banana-glucose tolerance test

**Figure 5:**
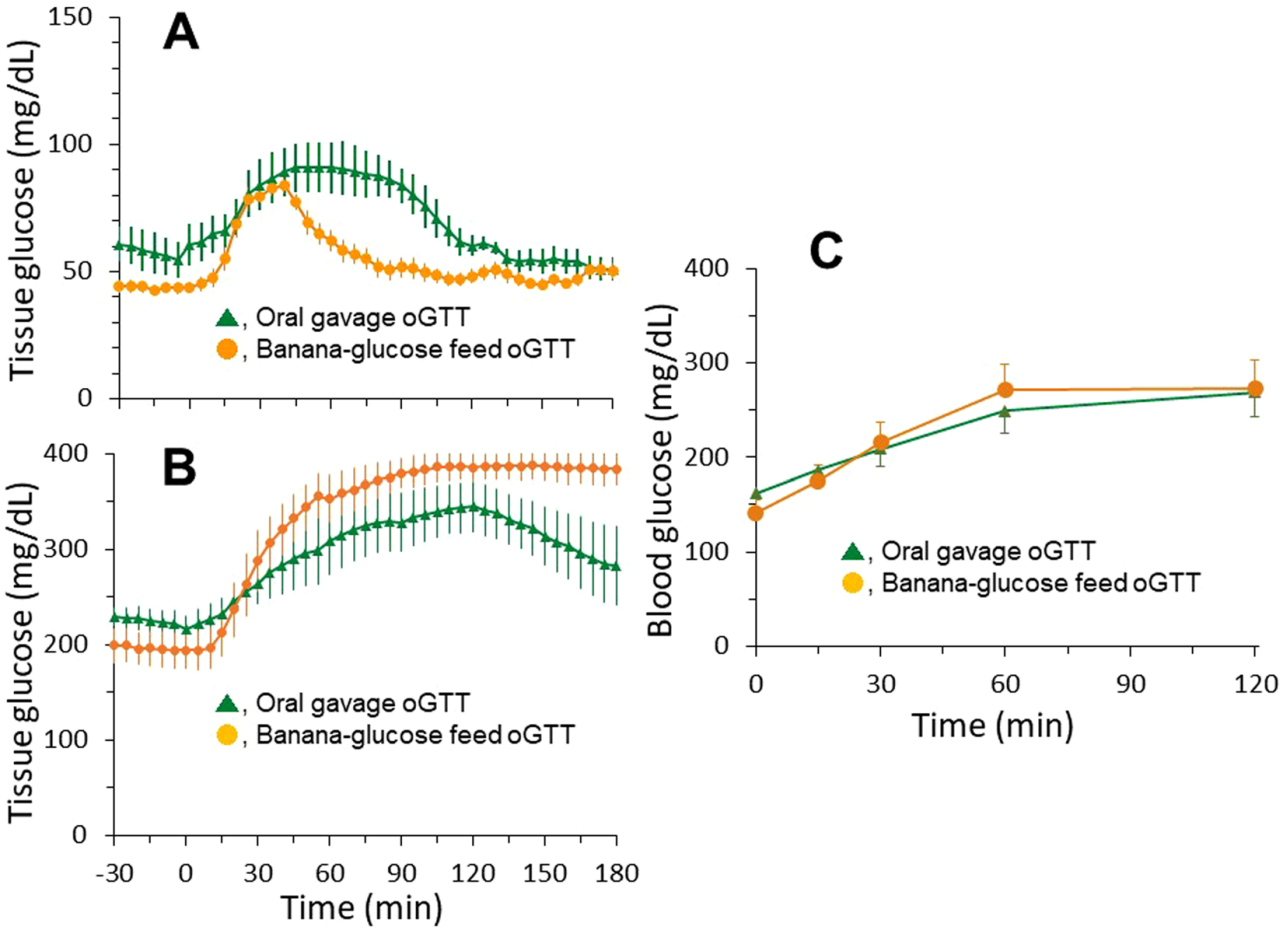
The tissue glucose changes during glucose-gavage oGTT and banana-glucose feed oGTT in the conscious normoglycemic (Panel A, n = 5) and hyperglycemic (Panel B, n = 5) monkeys. The interstitial glucose levels were continuously monitored by the implanted Dexcom G4 platinum devices. Panel C, The blood glucose changes measured by the glucometer during banana-glucose feed oGTT and glucose gavage oGTT in the conscious hyperglycemic monkeys (n = 24).

Classical oGTT in monkeys requires oral or nasal gavage of glucose when a monkey sits in monkey chair. Such gavage procedure itself can cause some stress and change of blood glucose^17, 22^. To test if oGTT could be conducted via a more natural way and avoid gavage-induced stress, a group of animals were implanted with Dexcom G4 Platinum. Each overnight-fasted experimental monkey with the glucose monitoring system stayed in its cage and was fed with a piece of banana (10 g/kg) packaged with glucose (1.75 g/kg). The changes of tissue glucose level were recorded and compared between banana-glucose feed oGTT and gavage oGTT in both normoglycemic (Fig. 5A, n = 5) and diabetic (Fig. 5B, n = 5) monkeys. The average baseline glucose levels in both normoglycemic and diabetic animals were approximately 20 mg/dL lower with the banana-glucose feed approach than with the classical gavage method (Fig. 5A,B). Compared with gavage oGTT, the hyperglycemic duration of banana-glucose feed oGTT was markedly shorter in the normoglycemic animals (Fig. 5A). The glucose level returned to the pre-oGTT level in 80 min after banana-glucose feed and in 120 min after glucose gavage in the normoglycemic animals. However, the hyperglycemic duration was prolonged with banana-glucose feed compared with glucose gavage in the diabetic animals (Fig. 5B).

The banana-glucose feed oGTT described above was used 10 g/kg banana plus 1.75 g/kg glucose per animal, but the gavage oGTT was administered 1.75 g/kg glucose only. To exclude the effect of the extra glucose in the banana on oGTT, the banana-glucose feed method was further validated. The sugar level in banana was analyzed by Pocket Refractometer (Model: PAL-RI, Atago, Tokyo, Japan, www.atago.net) and found the ratio of 10 g banana containing approximately 1 g sugar. An experiment was thus conducted in 24 diabetic cynomolgus monkeys with all the similar conditions and procedures, except classical oGTT via gavaging and banana-glucose oGTT via natural feeding. The overnight fasted monkeys were restrained in monkey chair and fed with 10 g/kg banana plus 0.75 g/kg glucose (equivalent a total of 1.75 g/kg sugar) or given by gavage with 1.75 g/kg glucose. The blood glucose levels were measured by glucometer immediately before and 15, 30, 60, 120 min after banana-glucose or glucose only administration. The blood glucose response curves were almost overlapped for banana-glucose feed oGTT and gavage oGTT (Fig. 5C). Again, the baseline glucose level (0 min) was 21 mg/dL lower with banana-glucose feed than with glucose gavage.

## Discussion

In this study two continuous glucose monitoring systems were validated and compared in nonhuman primates. Our data show that interstitial or blood glucose levels in conscious, stress-free cynomolgus monkeys could be accurately measured and recorded by the implanted Dexcom G4 Platinum or HD-XG telemetry system. The methods could monitor and record instant changes of interstitial or blood glucose. The interstitial or blood glucose parameters collected by the telemetry methods correlated significantly well with the readings by the classical glucometer test (Fig. 1). The advantages of glucose monitoring by the implantable telemetry devices in NHPs include: (1) 24-hr consecutive monitoring of glucose changes for a period of time from 7 days to 8 weeks^17^; (2) stress-free during glucose monitoring; (3) no anesthesia during GTT; (4) less labor intensity during GTT; (5) instant glucose readings without blood collection and assay. Our study demonstrates that remote and continuous glucose monitoring via implanted Dexcom G4 Platinum or HD-XG telemetry device in conscious, stress-free and moving-free monkeys is feasible and provides a sophisticated approach to investigate glucose changes due to daily activities, neuronal and hormonal changes, or physical/chemical challenges. Continuous glucose monitoring can avoid missing data points due to infrequent measurements by glucometer or blood sampling and provide timely therapy for either hyperglycemia or hypoglycemia clinically. In fact, one recent clinical trial in 161 adults with type 1 diabetes treated with multiple daily insulin injections showed continuous glucose monitoring resulted in better glycemic control compared with conventional treatment^23^. Participated patients had mean HbA1c 7.92% and were randomized to receive treatment using a continuous glucose monitoring system or conventional treatment for 26 weeks. The use of continuous glucose monitoring compared with conventional treatment resulted in lower HbA1c^23^. No user-calibration (factory calibrated) glucose sensors are also in market currently for clinically professional use and can reduce the hassle, painful and cost to diabetic patient, which will play an important role in diabetes management^24^.

This study used the Dexcom G4 Platinum system (Dexcom, Inc., San Diego, CA, USA) to monitor the changes of interstitial glucose in NHPs. This FDA-approved glucose monitoring system has been used mainly in diabetic patients and is able to sense interstitial glucose frequently to inform the timing and dosage of exogenous insulin delivery^25^. Compared with the arterial glucose telemetry monitoring method (HD-XG telemetry), the advantages of the Dexcom G4 Platinum system (subcutaneously) are relatively easier to install, less invasive, less expensive, and well acceptable from the safety profile which becomes the mainstay for patient use. The disadvantages of Dexcom G4 Platinum can firstly manifest moderately delayed glucose readings (5 to 12 min)^26–29^, especially to sudden glucose change, such as ivGTT, and likely worsen with implantation time as the encapsulation develops^30, 31^. Secondly, the Dexcom G4 Platinum system readings of interstitial glucose level can be various due to local fluctuations of subcutaneous blood flow by the change of temperature and/or mechanical pressure^32, 33^ and local glucose metabolizing. These factors plus time delay, especially during acute change of blood glucose of ivGTT or oGTT, could result in glucose levels lower in tissue than in blood (Fig. 3A,C). Therefore, such differences between glucometer measurements and Dexcom G4 readings were unlikely due to under-reading glucose levels by Dexcom G4, similar to the data showed in ivGTT measurements (Fig. 4A,C). Thirdly, subcutaneously inflammatory response results in biofouling and encapsulation, which limits sensor life (generally 2 weeks)^26, 34^. Fourthly, fixation of subcutaneously inserted sensor for stable data collection in physically active monkeys is challenging. In contrast, the HD-XG telemetry system can stabilize an implanted sensor in an artery and stably provide almost instant glucose readings^16^. However, compared with Dexcom G4 Platinum subcutaneous placement, the HD-XG telemetry method is relatively more invasive as the sensor has to be placed in a main artery. In addition, HD-XG telemetry device is currently used only in animal research and device itself costs much more than Dexcom G4 Platinum. The limited precious resource of diabetic NHPs plus relatively expensive HD-XG device caused the limitation of the study with a small sample size in HD-XG diabetic group (n = 2). Such sample size was difficult to run statistical analysis (Table 1). Fortunately, the current study is more qualitative and individual sample data are thus presented (Table 1, Figs 3D and 4D). Of course, further study with more animals is required if statistical significance needs to be granted. To make some comparisons between the two monitoring systems used in this study, their general characteristics are summarized (Table 2).

**Table 2:** General characteristics of two continuous glucose monitoring systems.

Device	Dealer	Sensor placed	Glucose tested	Sampling frequency	Sensor lasting	Main application	Cost
Dexcom G4 platinum	Dexcom Inc.	S.C.	Tissue fluid	Once/5 min	7 days	Human (FDA approved)	Low
HD-XG transmitter	DSI	Artery	Arterial blood	Once/10 sec	8 weeks	animal	Moderate

Note: DSI, Data Sciences International; S.C., subcutaneously.

Oral or nasal gavage is a typical way for glucose administration if an oGTT is performed in lab animals, including NHPs. The gavage procedure itself in monkeys could induce an obvious stress which affected blood glucose level^17^. Banana-glucose feed for oGTT in NHPs seems significantly reduced the stress level. The baseline glucose level was approximately 20 mg/dL lower in the banana-glucose feeding animals than in those oral glucose gavage ones (Fig. 5). Oral gavage, the most widely used method in lab animals, refers to the way that drugs or chemicals are dosed by putting a tube down animal throats to deliver substances directly to the stomach. It has been used for decades and offered precise dose and timing control. One study suggests oral gavage should be abandoned for hazard assessments of endocrine disrupting chemicals, because it is stressful with manual restraint in conscious NHPs^35, 36^ and interferes with endocrine responses^37^. Oral gavage, especially long-term^38^, can carry well-known risks, including respiratory interference, damage to the stomach or esophagus, and granulomatous inflammation^36, 39^, and potentially confound experimental outcomes^40^. Less than 1 percent of administered bisphenol A was bioavailable in blood in a gavage experiment with monkeys, while bisphenol A fed in a piece of fruit resulted in over 7 percent of administered bisphenol A being bioavailable in blood^37^. Alternative ways can reduce gavage-induced stress, such as feeding a compound mixed with a solution, food and pill^37, 38, 41^ or giving a compound via implanted devices, such as osmotic pump. In this study banana-glucose feed created a nature way for oGTT in NHPs accepted glucose voluntarily. The approach described here is convenient and effective for oral administration of glucose in conscious cynomolgus monkeys, which is obviously no stress for oGTT if glucose level is monitored by a continuous glucose monitoring system.

In summary, the Dexcom G4 Platinum and HD-XG telemetry systems were successfully used for continuous glucose monitoring in conscious, moving-free NHPs. These novel technologies allow researchers to monitor glucose changes during mechanistic research and drug discovery. Their advantages and disadvantages were compared and discussed. In addition, a natural approach without gavage-induced stress and other risks to run oGTT was validated via banana-glucose feed in conscious normoglycemic and diabetic NHPs.

### Declarations

#### Research involving human participants and/or animals

This study did not involve humans, but was conducted in monkeys. The study protocol and experimental procedures used in this study were approved by the IACUC of Crown Bioscience Inc., which includes member from outside of the company. The approval numbers are AN-1308-016-19 and AN-1507-004.

#### Informed consent

All the authors have read and approved the manuscript for submission. Their consents are available if requested.

### Availability of data and material

All the materials and relevant raw data supporting our findings can be found in Tables and Figures in the manuscript and are freely available to readers or scientists wishing to use them for non-commercial purposes.
